# Nutritional Profile of Children, Adolescents, and Women Living in Areas Potentially Affected by Heavy Metal Pollutants from the Revuè River in the Manica Province, Mozambique

**DOI:** 10.3390/epidemiologia7040103

**Published:** 2026-07-16

**Authors:** Tatiana J. Marrufo, Osvaldo F. Inlamea, Márcia I. Xavier, Celso M. Cumbula, Filipe Murgorgo, Cristovão Pereira, Ivalda Macicame, Bettencourt P. S. Capece, Maria-Raquel G. Silva

**Affiliations:** 1 Faculty of Science and Technology, University Fernando Pessoa, 4249-004 Porto, Portugal; 2 National Health Institute of Mozambique, Marracuene 020502, Maputo, Mozambique; osvaldo.inlamea@ins.gov.mz (O.F.I.); marcia.xavier@ins.gov.mz (M.I.X.); celso.cumbula@ins.gov.mz (C.M.C.); ivalda.macicame@ins.gov.mz (I.M.); 3 RISE-Health, Faculty of Health Sciences, Fernando Pessoa University, Fernando Pessoa Teaching and Culture Foundation, 4249-004 Porto, Portugal; raquel@ufp.edu.pt; 4 Research Centre of the Health Directorate, Chimoio 060601, Manica, Mozambique; murgorgo@gmail.com; 5 Faculty of Environmental Engineering and Natural Resources, Zambeze University, Chimoio 060603, Manica, Mozambique; cristovaopereiraq@gmail.com; 6 Faculty of Veterinary, Eduardo Mondlane University, Km 1.5, Maputo 010506, Mozambique; bpscapece@yahoo.com; 7 FP-I3ID, FP-BHS, Fernando Pessoa, Research, Innovation and Development Institute, Fernando Pessoa, Biomedical and Health Sciences Research Unit, Faculty of Health Sciences, University Fernando Pessoa, 4200-150 Porto, Portugal; 8 Molecular Oncology and Viral Pathology Group, Research Center of IPO-Porto (CI-IPOP) & RISE@CI-IPOP (Health Research Network), Portuguese Oncology Institute of Porto (IPO-Porto)/Porto Comprehensive Cancer Center Raquel Seruca (Porto. CCC), 4200-072 Porto, Portugal; 9 CHRC, Comprehensive Health Research Centre—Group of Sleep, Chronobiology and Sleep Disorders, Nova Medical School, Nova University of Lisbon, 1150-090 Lisbon, Portugal; 10 CIAS, Research Centre for Anthropology and Health—Group of Human Biology, Health and Society, University of Coimbra, 3000-456 Coimbra, Portugal

**Keywords:** nutritional status, children, adolescent, women, artisanal mining, heavy metals, Mozambique

## Abstract

Background: The global nutrition crisis continues to disproportionately affect vulnerable populations’ health in regions where artisanal mining is high. Objective: This study assessed the nutritional status of children, adolescents, and women living in the Revuè sub-basin, who are potentially exposed to heavy metal contaminants. Methods: An observational and cross-sectional design was used to combine anthropometric and sociodemographic data with laboratory analysis for the assessment of heavy metal contaminants in drinking water sources. Results: Results indicated a high prevalence of stunting among children under five (28.4% of boys and 19.9% of girls) while female adolescents were underweight (25.7%), particularly during the rainy season. In contrast, 19% of women were overweight and 3.1% were obese. A modified Poisson regression analysis revealed a significant negative association between mercury exposure (RR = 0.77, *p* = 0.02) and stunting in children, whereas higher educational attainment among husbands was protective for women (RR = 0.76, *p* = 0.03). The assessment of mercury exposure through water consumption showed statistically significant variations across regions and seasons (*p* < 0.001). Water analyses identified high concentrations of arsenic (0.03 mg/L, 95% CI: 0.02–0.10) and mercury (0.09 mg/L (95% CI: 0.003–0.009), exceeding safety standards. Conclusion: Overall, these findings highlight the nutritional and health risks potentially linked to artisanal mining.

## 1. Introduction

The global nutrition crisis is having a disproportionately adverse effect on people from various geographical regions, including vulnerable populations living in highly polluted areas. It is estimated that 155 million children worldwide suffer from stunting as one of the most prevalent forms of undernutrition, and nearly half of all deaths occur in children under five years of age [1]. Additionally, it is estimated that over one billion girls and women experience undernutrition, micronutrient deficiencies, and anemia, as well as maternal underweight, short maternal stature, and low birthweight. These factors have collectively been identified as predictors of stunting and wasting in early childhood [2].

Approximately 20% of the African population is undernourished. Of these people, 239 million live in Sub-Saharan Africa, where the burden of undernourishment varies between the eastern (133 million) and southern (5.3 million) regions [3]. Around 45% of those affected are children, which is a lower proportion than reported for adolescent girls and women in the UNICEF Global Nutrition Crisis report [2,4]. The national prevalence of chronic and acute malnutrition among children under five is 38% and 4%, respectively. Fifteen per cent of children are underweight, while 5% are overweight [5]. This varies across Mozambican provinces, wherein a range from 39 to 47 of women aged 20–49 years, 5% are underweight (body mass index [BMI] < 18.5 kg/m^2^), while 28% are overweight (BMI ≥ 25.0 kg/m^2^) or obese (BMI ≥ 30 kg/m^2^) [5]. Despite the high rates of undernutrition in Southern Africa, the agriculture and artisanal mining sectors remain major contributors to the local economy in the region, and are also major risk factors for their populations’ health, especially the most vulnerable groups, such as children, girls, and women [5].

Globally, environmental hazards account for over 24% of childhood deaths [6], highlighting the urgent need to address health outcomes in the population associated with both traditional (pathogens, pesticides, phosphates, nitrates, and heavy metals) and emerging environmental exposures [7]. Small-scale and artisanal mining pose significant environmental challenges, imposing serious health risks on miners and the surrounding communities [8,9]. Chemical contaminants released during mining activities enter the food chain via the soil, water, and sediments, and subsequently accumulate in crops and aquatic organisms. These contaminants bioaccumulate and biomagnify across trophic levels, ultimately reaching humans through systemic absorption and contributing to adverse health outcomes, particularly in populations dependent on contaminated water and food sources [9,10]. Nevertheless, only limited evidence has been found to demonstrate the relationship between chemical contaminants, such as heavy metals from artisanal mining, and the nutritional status of exposed populations [11,12].

In Southern Africa, where food scarcity is a major concern, food is a primary source of exposure to hazardous pollutants and toxins [11]. Specifically, fish and seafood are the primary sources of exposure to methylmercury, while arsenic in groundwater is a common cause of excess arsenic exposure through drinking water. Consequently, vulnerable groups residing in these areas are exposed daily to biotoxicity caused by heavy metals, particularly arsenic (As), cadmium (Cd), lead (Pb), and mercury (Hg) [12]. This has a detrimental effect on their growth, development, health, and well-being. Postnatal exposure to Pb has been demonstrated to be associated with impaired neurodevelopment in children [13], alterations in sex hormone concentrations, and adverse effects on female reproductive health [14]. In contrast, prenatal maternal exposure to Hg may have long-lasting consequences, including disruptions to neurodevelopment, behavior, cognitive functioning, motor skills, and the maturation of the immune and reproductive systems. Furthermore, exposure to mercury (Hg) may exacerbate malnutrition and micronutrient deficiencies in children, adolescents, and women due to the complex interactions between nutrients and toxic metals within biological systems [15,16].

Although research into the environmental crisis encompassing pollution, waste, biodiversity loss, and climate change is still emerging, high-volume discharges of pollutants are increasingly threatening aquatic and terrestrial ecosystems [16]. Their persistence, toxicity, and bioaccumulation facilitate infiltration into drinking water via urban, artisanal, and industrial waste. This contaminates soil and groundwater, compromising water quality, and exposes populations to significant risks [7]. Moreover, in 2025, Jiang et al. [17] noted that water contamination exacerbated global scarcity and undermined food security by reducing the availability of potable water for consumption and irrigation, with serious implications for agriculture and supply chains. Therefore, it is essential to investigate the potential environmental exposure to heavy metals and the nutritional status of these vulnerable individuals. Furthermore, the scientific literature on heavy metals as sources of environmental pollution in Mozambique remains very limited. Thus, the present study aimed to (i) determine the nutritional status of children, adolescents, and women attending health facilities located in the Revuè sub-basin, Manica (Mozambique); (ii) analyze the nutritional status of participants across health facilities during different seasons (dry and rainy); and (iii) assess and analyze the risk of water contamination through the characterization of heavy metal contaminants.

## 2. Materials and Methods

### 2.1. Study Design

This observational and cross-sectional study was conducted in Manica Province, Mozambique. The Revuè River sub-basin covers an estimated 4400 km^2^ and originates in the mountainous terrain of the Manica District. This area is characterized by the Manica Greenstone belt, which contains rich alluvial gold deposits. The river primarily flows through the Manica District and the adjacent districts, including Macate and Sussundenga [18]. The climate can be categorized into two distinct environmental seasons: the rainy season (from October to March of the following year) and the dry season (from April to September). Demographic data from these districts indicate that a substantial proportion of the population is under the age of 15 (48.7%), with females comprising more than half of the total population [19].

**Figure 1:**
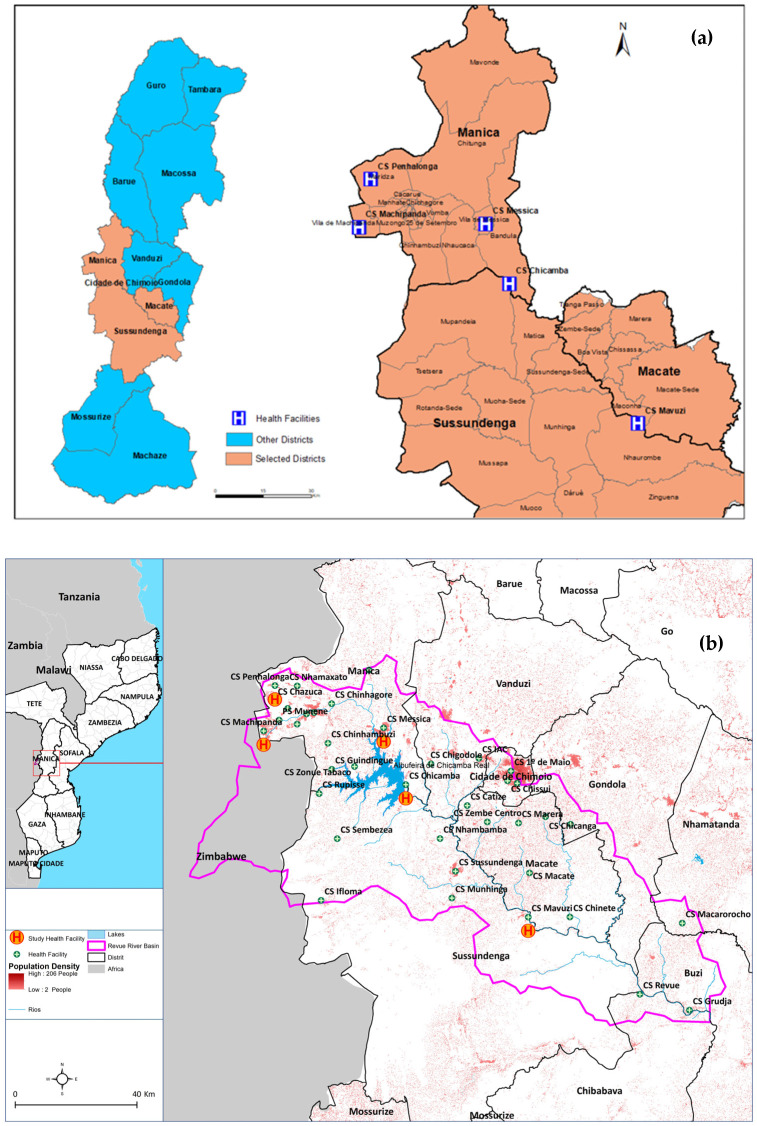
Study area in Manica Province. Part (**a**) shows the districts included in the study within the province and the health facilities located within the study area. Part (**b**) shows the dimension of the Revuè sub-basin and the five health facilities included in the study.

The study was conducted in 2025 across five health facilities located in the Revuè River sub-basin in Manica Province (Figure 1) during both rainy and dry seasons. Participants included children under five years old, children aged 5–11 years, adolescents aged 12–17 years, and women aged 18–49 years. They were recruited from the selected health facilities in Penhalonga, Machipanda, Messica (District of Manica), Chicamba (District of Sussundenga), and Mavuzi (District of Macate). These health facilities were chosen based on their geographical proximity to the Revuè River watercourse, following the methodological framework outlined by de Bakker et al. in 2021 [20]. Additionally, the sample design ensured that at least one sample from each community within the Revuè sub-basin health area was included [8].

### 2.2. Participants Recruitment and Data Collection

Participants were recruited from health facilities in the selected area. Individuals who regularly attended appointments for follow-up care, preventive services, maternal and child health services, and other essential services were eligible for inclusion in the study. Participants were approached during their regular medical visits to the health facility. They were provided with detailed information about the study and the recruitment procedures. Before participating in the study, patients were given an informed consent form that clearly set out the study’s objectives, the procedures to be performed, the potential risks and benefits, and the participants’ rights. The present study exclusively included voluntary participants. Mothers who voluntarily participated in the study and were carrying children within the age range of the study’s inclusion criteria were interviewed using a children’s questionnaire. A questionnaire specifically designed for women was administered for those seeking care at the health facility. The inclusion of mother–child pairs in the study was contingent upon both individuals visiting the health facility for any available healthcare service.

The recruitment of participants was conducted by trained health professionals, who employed the following inclusion criteria: (1) children and adolescents aged 1–17 years; (2) women aged 18–49 years; (3) residence within the study area and a regular user of the health facility; (4) participants who were physically and mentally capable of participating in the interview, and (5) the capacity to provide informed consent, either by adult participants or the legal representatives of children and adolescents.

For each participant, a semi-structured questionnaire was administered to collect sociodemographic information, general health status, nutrition history, and anthropometric measurements. Furthermore, the questions were aligned with national surveys conducted in the country to assess nutritional indicators and feeding habits of children and women [21,22] ensuring that the questions had been adapted to suit the cultural context.

### 2.3. Ethical Approval

All participants provided informed consent. Parental consent was obtained for all the children and adolescents included in the study. However, additional assent was required from children aged between 12 and 17 years. The ethical approval for conducting this research was granted by the Mozambique Committee of Bioethics in Health under the reference number 024/CNBS/24. The collection of all data was conducted in accordance with national regulations and the Declaration of Helsinki [23].

### 2.4. Variables

Sociodemographic data collected from children and adolescents included variables such as gender, age, nutritional history, season, and region of data collection. Health-related variables were also assessed, including dietary habits among children and adolescents, as well as their parents’ nutritional status. The nutritional history encompassed information on breastfeeding practices and the introduction of complementary foods. For all parents accompanying children, nutritional status was assessed measuring their anthropometrics to determine BMI. For women, in addition to age and educational background of their spouse, data on family income issues were also collected, along with the season and region of data collection.

Anthropometric measurements included weight, height, head circumference, and mid-upper arm circumference (MUAC). Participants’ body weights were measured using a dry scale with an accuracy of 100 g and a digital display. Head circumferences and MUAC were measured with a non-extensible tape that was assessed in children up to five years of age. For this group, indicators related to MUAC, weight-for-age (WA), and weight-for-height (WH) indices were assessed according to Fink et al., 2024, and Zaba et al., 2022 [24,25]. For children under five years (0–59 months), z-scores were calculated using the WHO Child Growth Standards (2006) [1]. Standardized indicators, such as weight-for-age (WA), height/length-for-age (HA), weight-for-height/length (WH), and BMI-for-age (BA), were generated using the WHO SPSS macro (igrowup.sps). Children were classified according to WHO cut-offs as follows: (1) stunting—HA < −2 SD; (2) wasting—WH < −2 SD; (3) underweight—WA < −2 SD; and (4) overweight—BA > +2 SD.

For adolescents, indicators of underweight, overweight, and obesity were determined by converting measurements into HA, WA, and WH z-scores based on the WHO Child Growth Standards [1,26]. For children and adolescents, BMI-for-age was calculated in Statistical Package for Social Science (SPSS) using the WHO 2007 Growth Reference to generate z-scores. Cut-offs were interpreted as follows: (a) underweight, <−2 SD; (b) severe underweight, <−3 SD; (c) overweight, >+1 SD (approximately equivalent to a BMI of 25 kg/m^2^); and (d) obesity, >+2 SD (approximately equivalent to a BMI of BMI 30 kg/m^2^). For women, BMI was calculated using weight (in kilograms) and height (in meters) and categorized into four BMI groups [1]: underweight (<18.5 kg/m^2^), normal weight (≥18.5 and ≤24.9 kg/m^2^), overweight (≥25.0 and ≤29.9 kg/m^2^), and obesity (≥30.0 kg/m^2^).

For water collection, the following criteria were employed: (i) near the source of the Revuè River (upstream) in Penhalonga; (ii) in a populated area through which the river passes, at the level of the main town of Messica (both upstream and downstream); (iii) in the Chicamba reservoir (upstream and downstream), and (iv) near the mouth of the Revuè River, where it flows into the Búzi River at the Mavuzi reservoir (downstream). All sampling points were accurately georeferenced using Geographic Positioning System (GPS) devices to record latitude and longitude data during both the dry and rainy seasons. The water samples collected included three different depths: surface river water, deep river water, and groundwater (two points each). Collected water is used for population consumption. Sampling was conducted at four points along the Revuè River: (i) Penhalonga (P1)—latitude −18.8669087, longitude 32.7798868, altitude 799 m; (ii) Messica (P2)—latitude −18.9744927, longitude 33.0507333, altitude 622 m; (iii) Chicamba (P3)—latitude −19.1718892, longitude 33.1401906, altitude 626 m; and (iv) Mavuzi (P4)—latitude −19.5269405, longitude 33.4924137, altitude 346 m.

Furthermore, trained research technicians collected, handled, and stored environmental samples following the guidelines recommended by APHA et al., 2017 [27]. Water samples were collected using blank sampling techniques and replicate samples were taken for the determination of heavy metals (As, Cd, Hg, and Pb) in polypropylene plastic bottles and conical tubes. The samples were tested in situ with the following parameters: temperature (T°), turbidity, and pH. Subsequently, the samples were preserved with nitric acid (HNO3) at a ratio of 1.5 milliliters HNO_3_ per 1 L of the sample. For the analysis of dissolved metals, the samples were transported in isothermal boxes to the laboratory. Heavy metal analysis was conducted using the method of Anodic Stripping Voltammetry (ASV) with a Metalyser Portable HM1000 (Trace2O Ltd., Berkshire, UK) [28,29]. This method has been validated nationally for in situ environmental monitoring, with its performance benchmarked against conventional laboratory assays [30,31]. Data interpretations were performed by comparing the results obtained with established water quality standards [1,32].

### 2.5. Sample Size

A stratified random sampling technique was employed, with the different sample groups (children, adolescents, and women) being considered based on the district’s population by age group according to the National Institute of Statistics (INE, 2017) [19]. The sample size was determined by employing the OpenEpi statistical software, version 3. In this calculation, the expected prevalence was set at 50% and the significance level at 5%. The design effect for cluster surveys was also considered. The total number of participants per age group to be recruited for the study was estimated, and this approach was used to recruit participants per health facility. This approach involved individuals (children, adolescents, and women) who attended the selected health facilities and met the selection criteria during the study period (February to December 2025) and were therefore included in the sample.

### 2.6. Statistical Analysis

Data analysis was performed using SPSS (version 30.0 for Windows). Normality was assessed using the Shapiro–Wilk test. Comparisons of mean anthropometric values between age groups (children, adolescents, and women) and across seasons (rainy and dry) were conducted using two-way ANOVA. When the assumption of normality was not met, Kruskal–Wallis’s test was employed to assess differences in nutritional status among children, adolescents, and women.

Anthropometric indicators were standardized using the WHO Child Growth Standards (2006) for children under five and the WHO Growth Reference (2007) [1] for adolescents. BA and HA z-scores were calculated with WHO macros. Prevalent nutritional outcomes, stunting in children under five, and overweight in women were modeled as binary dependent variables. Associations with age, gender, education, nutrition history, family income, and environmental exposures were examined using multivariate logistic regression. We estimated multivariable risk ratios and risk differences using generalized linear models in SPSS. First, we attempted to fit a log-binomial model using the GENLIN procedure [33] with a binomial distribution and log link to obtain adjusted risk ratios. Because log-binomial models are known to exhibit convergence difficulties, we applied the modified Poisson approach with robust variance. In this specification, the outcome was modeled using a Poisson distribution with a log link. The models employed followed the structure of Huh et al. [34]. Risk ratios (RRs), Risk differences (RDs), and confidence intervals (CIs) were reported.

**Figure 2:**
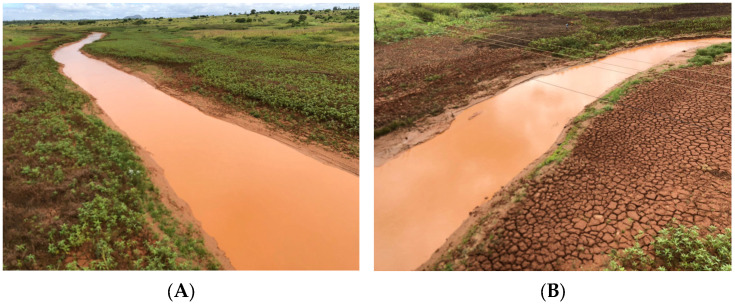
Revuè River with high levels of turbidity observed in both seasons: (**A**) rainy and (**B**) dry. The picture was taken by the authors in the study area.

The assessment of environmental exposure to heavy metals was conducted using water samples collected from the study river. It should be acknowledged that exposure pathways in artisanal mining areas are complex. The present study therefore assessed water samples, given the high levels of turbidity observed in the local context (not measured) at both seasons (Figure 2). The measured concentrations of heavy metals were compared with the national water quality standards using a one-sample test. With this test, we evaluated whether the mean concentrations of each metal in the collected samples seasonally differed significantly from the reference values established by national and international guidelines. The analysis was performed separately for each metal (As, Cd, Hg, and Pb) and water sampling source (surface, deep river, and groundwater) across the dry and rainy seasons and then combined with mean global concentrations. The results obtained are presented as mean values, along with the corresponding *p*-value for seasonal variation.

According to Kayanni and Mohammed, 2025, and Posthuma et al., 2002 [35,36], the population-level risk of exposure was estimated through population attributable fractions (PAFs), calculated from exposure prevalence derived from the analyzed water samples. The calculation of daily exposure doses for detected heavy metals was based on three factors: water concentration, estimated water intake by age, and measured body weight. Hazard quotients (HQs) were subsequently derived by dividing the estimated intake dose by the reference dose (RfD), thereby providing an indication of potential health risks, which can be expressed as the relative risk (RR). The prevalence of risk was then calculated for all age groups that were statistically significant in relation to exposure outcomes across different geographical areas. The statistical significance of the findings was established at *p* < 0.05.

## 3. Results

### 3.1. Participants’ Characteristics

A total of 1098 individuals meeting the selection criteria were included in the study and accordingly distributed across five health facilities: Penhalonga (n = 106; 9.7%), Messica (n = 297; 27.0%), Chicamba (n = 156; 14.2%), Machipanda (n = 143; 13.0%), and Mavuzi (n = 396; 36.1%). Among the total participants (n = 1098), approximately 34.1% (n = 345) of the children and adolescents recruited for the study were under five years old, of which 316 were girls (53.8%) and 271 boys (46.2%) (Table 1). About 80.0% of children and adolescents (n = 470) were breastfed for less than 12 months, and 49.4% (n = 290) were introduced to complementary foods before reaching six months of age. Additionally, 368 women (72.2%) and 389 of their husbands (87.2%) reported being able to read. For 33.3% of the evaluated families, agriculture was the primary source of economic activity. Furthermore, 395 women (77.2%) lived on a minimum wage. Most of participants were recruited from the Mavuzi health center in both groups (children and adolescents, 42.1%; women, 29.2%), primarily during the rainy season (children and adolescents, 63.9%; women, 62.7%) (Table 1).

### 3.2. Nutritional Status

#### 3.2.1. Children Under Five Years Old

**Figure 3:**
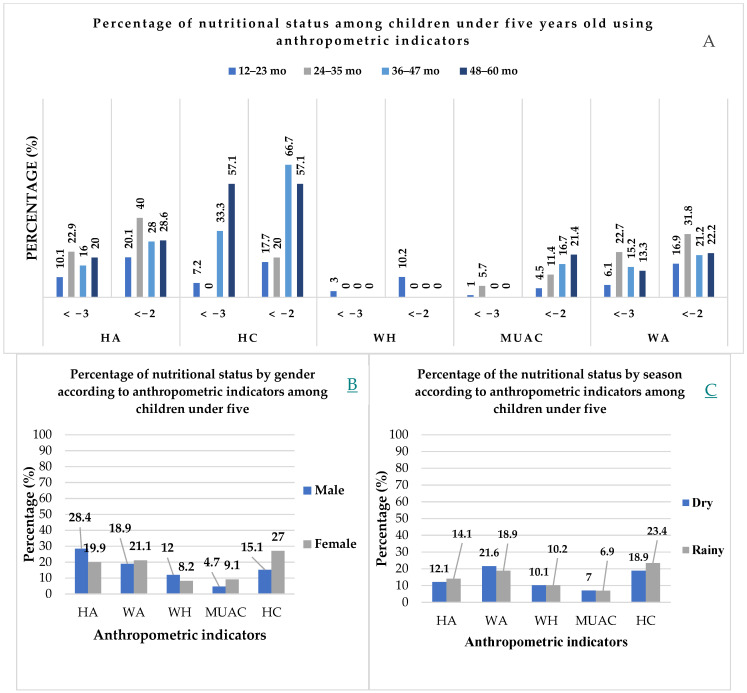
Percentage of nutritional status (less than two and three standard deviations) among children under five years old, disaggregated by age in months (**A**), gender (**B**), and season (**C**) according to z-scores: height-for -age (HA), weight-for -age (WA), weight- the-height (WH), middle-upper arm circumference-for-the-age (MUAC), and head circumference-for -age (HC). Abbreviation: mo—months.

Anthropometric indicators were summarized by sample size (n), prevalence of severe outcomes (<−3 SD), moderate outcomes (<−2 SD), and mean ± SD z-scores (Table 2). The z-scores of indicators HA, WA, WH, MUAC, and HC are presented by age group, gender, and season. Across indicators and subgroups, stunting (HA) emerges as the prevalent nutritional outcome; underweight (WA) is present but generally less prevalent than stunting in the age group results. Stunting peaked in the 24–35 months group (40.0% SD < −2: mean −1.1 ± 2.4) compared to the other age groups. HC z-score showed deficits in the 12–23 months (17.7% SD < −2: mean −0.6 ± 1.5) and 24–35 months (20.0% SD < −2: mean −0.5 ± 1.8) groups, with much higher values in the 36–47 months and 48–60 months groups. MUAC deficits increased with age from 12–23 months (4.5% SD < −2: mean +0.2 ± 1.3) to 48–60 months (21.4% SD < −2: mean −0.7 ± 1.1). The prevalence of underweight was high in the 24–35 months group (31.8% SD < −2: mean −1.2 ± 2.2) and remained high in 36–47 months and 48–60 months groups. The prevalence of wasting was reported exclusively for the 12–23 months age group (10.2% SD < −2; 3.0% SD < −3), as illustrated in Figure 3A.

A comparative analysis of the prevalence of height-for -age across both sexes revealed some differences (Figure 3B). The observed percentages were marginally higher in males (28.4%) compared to females (19.9%). The observed percentage of weight-for- age subjects was also comparable, being lower in males (18.9%) than in females (21.1%). In contrast, the prevalence of weight-for- height was found to be higher in males (12%) compared to the prevalence of MUAC and HC deficits, which were observed to be higher in females than in males, with observed percentages of 9.1 and 27.0%, respectively.

Seasonal fluctuations in anthropometric indicators among children under five years of age (see Figure 3C) indicated that 21.6% of children exhibited a weight-for-age z-score < −2 SD during the dry season, as compared to 18.9% during the rainy season. The prevalence of HA was observed to be 12.1% in children during the dry season, which was marginally lower than the 14.1% observed during the rainy season. In both seasons, the distribution of WH and MUAC demonstrated the proportion of households with HC was marginally elevated during the rainy season (23.4%) in comparison to the dry season (18.9%).

#### 3.2.2. Children and Adolescents

For children and adolescents, nutritional status based on BMI-for-age is presented by sex and season (Table 3). Among adolescents (aged 12–17 years), underweight was the prevalent outcome in both sexes, with 20.3% of males and 25.7% of females exhibiting a BMI-for-age < −2 SD. Approximately 8.6% of girls and 8.5% of boys were overweight (BMI-for-age > +1 SD), while obesity was less common (<3.4%). Boys showed a higher prevalence of overweight at younger ages (6–11 years), whereas girls exhibited greater underweight during mid-adolescence (12–17 years). The mean BMI-for-age z-scores were found to be negative for both sexes (males: −0.8 ± 1.4; females: −1.0 ± 1.6). Among all 164 children and adolescents, BMI-for-age z-scores indicated a high prevalence of underweight (23.8%), as well as severe underweight (10.4%), across both dry and rainy seasons. Severe underweight was more prevalent during the rainy season (13.9%) compared to the dry season (3.6%). Overweight also affected this age group (8.5%), particularly during the dry season (16.1%) compared to the rainy season (4.6%).

#### 3.2.3. Women

The nutritional status of women is presented in Table 4. The principal nutritional outcome emphasized in these findings was the prevalence of overweight and obesity, which was higher during the rainy season (60.7%, n = 65) compared to the dry season (39.3%, n = 42). A higher prevalence of underweight women was observed during the rainy season (n = 20) in comparison to the dry season (n = 2), although this small size should be interpreted with caution. Most women across all regions exhibited a normal BMI in both seasons (rainy and dry, 61.4% vs. 38.6%, respectively).

### 3.3. Risk Factors Associated with Nutritional Status of Children Under Five and Women

A multivariable regression analysis was conducted to identify sociodemographic and environmental factors associated with stunting among children under five years old (Table 5). The variables analyzed included: child gender, duration of breastfeeding, timing of introduction of complementary food, parental BMI, season, and environmental exposure to arsenic and mercury. The final adjusted models were found to include the following variables: gender, the timing of complementary food introduction, and exposure to arsenic and mercury. Mercury exposure was a significant predictor in both models, namely in log-binomial regression (RRa = 0.76, 95% CI: 0.62–0.94, *p* = 0.01), and in the adjusted model (RD = 0.77, 95% CI: 0.63–0.95, *p* = 0.02), demonstrating an unexpected negative association. The variables of gender, arsenic exposure, and food introduction were found to be non-statistically associated.

In the case of women, the following variables were considered in the model: level of education, income source, family income, season, and environmental exposure risk. In the final adjusted model, the variables that remained were women’s and their husbands’ ability to read, family income allocated for food, and exposure risk to arsenic and mercury. A husband’s inability to read was significantly associated with a reduced risk of being overweight in both models, namely the adjusted log-binomial regression (RRa = 0.74, 95% CI: 0.58–0.95, *p* = 0.02) and in the risk difference model (RD = 0.75, 95% CI: 0.59–0.96, *p* = 0.03), as per Table 6. Maternal literacy, household food expenditure, and environmental exposures (As and Hg) demonstrated an absence of consistent influence. The findings of this study indicate that the probability of being overweight in this particular dataset is more responsive to sociodemographic factors than to environmental exposure.

### 3.4. Water Determination of Heavy Metals

The quality of water parameters in the Revuè sub-basin exhibited variation across sampling sites and depths during both dry and rainy seasons (Table 7). This variation was determined in regions where the population is exposed to mining activities through water intake and alongside the food chain. A total of 30 water samples were collected per season across four sampling sites and at three different depths, with three replicates per sample. The limits of detection and quantification were established in accordance with established national and international standards. The calibration and quality control procedures were grounded in the local context using laboratory-validated techniques. These techniques comprised strategies to confirm results through repetition and the utilization of replicates to manage values below detection limits. Water temperature exhibited seasonal variation, with slightly higher values recorded during the rainy season (25.7–28.0 °C) compared to the dry season (24.0–28.3 °C). Furthermore, the pH values remained within WHO and nationally recommended ranges (6.5–8.5) at all sites in both seasons, although sites in Chicamba and Mavuzi showed slightly higher pH during the rainy season (7.7). Turbidity exhibited the most pronounced spatial and seasonal variability. During the dry season, turbidity levels ranged from a minimal level at Messica and Mavuzi (0.2–2.6 NTU) to remarkably elevated values at Penhalonga and Chicamba (160.0–864.0 NTU). A similar pattern was observed in the rainy season, with turbidity again exceeding WHO and national limits of 5 NTU at multiple sites, particularly Penhalonga and Chicamba (51.5–807.0 NTU).

The mean concentrations for arsenic (in the river), cadmium, mercury, and lead were all found to be at the threshold of the National Standards. However, the mean concentration of mercury was found to be above WHO international standards. All parameters (with the exception for arsenic in groundwater) demonstrated a *p*-value of <0.001 for seasonal variability (Table 8). Furthermore, while the presence of arsenic was established in river water, it was found to be below the level of detection in groundwater. In the cases of cadmium and lead, both elements were found to be at the maximum allowable limits (0.003 mg/L and 0.01 mg/L, respectively) across all sources.

### 3.5. Population at Risk of Exposure to Heavy Metals

**Figure 4:**
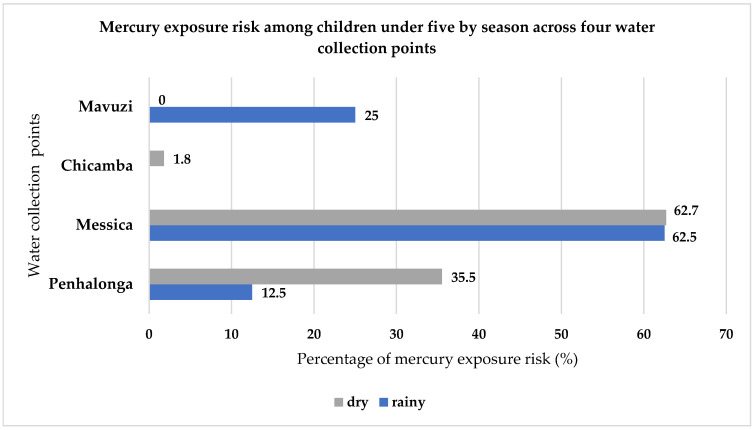
Percentage of exposure risk to the heavy metal mercury among the studied children under five in the Revuè sub-basin in both dry and rainy seasons.

The risk of exposure to mercury across the four sites in both the rainy and dry seasons is illustrated in Figure 4. Among children under five, assuming exposure of the population occurs through water ingestion, daily exposure doses for mercury were calculated based on water concentration, estimated water intake by age, and measured body weight. The following formula was utilized to derive the dose of Hg: Dose_Hg = (Hg_ugL × water_Lday)/weight_kg. Hazard quotients (HQs) were derived by dividing the Hg dose by the reference dose (RfD) using the following formula: HQ_Hg = Dose_Hg/RfD_Hg.; in which, RfD is used as stipulated in the national guidelines [33]. This quotient is representative of the relative risk (RR) of a potential outcome, with an HQ greater than 1 indicating potential health risk. The binary risk indicators (HQ > 1) were utilized to estimate the proportion of the study population at risk of exposure to mercury levels that exceed the safe threshold. Notwithstanding the constraints inherent to the utilization of data regarding the national population and mining activities, during the dry season, mercury exposure risk in this age group appears to be non-existent across Chicamba, indicating a markedly localized contamination pattern between seasons and regions. The present findings suggest that the risk of exposure to mercury is widespread in all sites, with the highest levels recorded in Messica at both seasons. During the rainy season, the lowest risk was reported in Penhalonga (12.5%), while during the dry season, this was reported in Chicamba (1.8%).

## 4. Discussion

This study was conducted in Mozambique’s Revuè sub-basin to evaluate the nutritional status of children under five, adolescents, and women living in an artisanal mining area. This area is potentially exposed to heavy metal discharges from mining activities into the river and groundwater. The present study provides evidence relating to the nutritional status of certain vulnerable groups in neighborhoods where mining activities are prevalent. These findings provide insight into how surrounding factors may influence the health of these target groups. Among the participants, 26.5% of children under five years of age were stunted; 23.8% of young children and adolescents, aged 6 to 17 years, were underweight; and 21.3% of women, aged 18 to 49 years, were overweight. The present study’s findings demonstrated an anthropometric profile indicative of undernutrition among the young children and adolescents included, as well as a transition to overweight among women. According to the 2022–2023 Mozambican DHS [21], these figures are marginally below the provincial averages (35% stunting, 11% underweight children, and 23% overweight and obese women) and the national averages (38% stunting, 13% underweight children, and 25% overweight women). Other studies conducted in Mozambique have documented a contrast in malnutrition outcomes, where levels were above the national average (with a prevalence rate of up to 40%) [24,38,39,40,41]. Similar results were observed in Sub-Saharan Africa and South Asia [4,42], underscoring the coexistence of chronic undernutrition in children alongside underweight and overweight in women. This reflects the cumulative effects of food insecurity [3]. The persistence of stunting and underweight among children in our study area indicates long-term growth deficits, while the increasing prevalence of overweight among women suggests an emerging risk of diet-related non-communicable diseases. These patterns together emphasize the need for integrated interventions that address both undernutrition and overweight simultaneously, thereby strengthening resilience across generations. Although gender and the early introduction of complementary foods (before six months) were not significant predictors of stunting in our study area, Garcia et al. [38] reported them as relevant associated factors in a Mozambican setting. Nevertheless, environmental exposure to heavy metals was associated with children’s nutritional status from a very young age, highlighting the importance of community-based, integrated interventions for children’s growth and nutrition [41,43]. Although the studied population was predominantly female, stunting and wasting were prevalent among male children under five. This pattern has been observed in other studies [1,25,40,44]. This may be due to biological susceptibility to infections in early life, suggesting that boys may have a weaker immune response and be more vulnerable to nutritional deficits in infancy than girls. In contrast, our study found that female adolescents were more affected by underweight and overweight than males, emphasizing that girls face unique nutritional challenges during puberty, in early pregnancies, and with dietary restrictions, particularly in low- and middle-income country settings [45]. The study also showed that girls approaching reproductive age were at a higher risk of being overweight or obese, which suggests long-term implications for maternal health. These findings emphasize the importance of monitoring childhood nutrition programs [39] by implementing age- and gender-sensitive interventions to reduce inequalities and empower girls by providing them with access to diverse diets and improved nutrition services. In Africa, artisanal and small-scale gold mining (ASGM) is a significant source of mercury and other toxic elements, such as arsenic, cadmium, and lead, which contaminate water, soil, and food systems [5,9]. This increases exposure among miners, their families, and nearby communities. As Cossa et al. [41] have asserted, such contamination has been linked to several health risks, including stunting in children, underweight in adolescents, and metabolic risks in adults, particularly in riverine and groundwater-dependent settings. However, the present study did not consider geographical issues or socioeconomic factors that were not controlled for. The study identified turbidity and mercury as factors contributing to the deterioration of water quality and the potential for public health risks. As demonstrated in previous studies [8,9], it can be hypothesized that seasonal variation may have influenced the risks observed. This is due to reduced water volume and elevated metal concentrations during the dry season, while increased tributary flow during the rainy season serves to dilute metals but increase turbidity through sediment transport. Our findings confirmed this hypothesis, as contaminant levels were found to be exactly at the limit of the high-risk scenario, where any fluctuations could potentially lead to toxicological levels. Although heavy metal concentrations were not stable throughout the year in our study, in mining-impacted areas, this may mask dangerous seasonal peaks above acceptable levels, as reported in other studies [46,47]. In addition, the borderline compliance of river water with national mercury and lead standards, coupled with high seasonal variability, suggests that children in the Revuè sub-basin are intermittently exposed to concentrations that exceed safety thresholds. This may exacerbate the nutritional vulnerabilities identified in this study. These findings highlight the importance of seasonal monitoring, as precipitation appears to affect the release of certain heavy metals into river systems.

Given the intricate interplay between the socioeconomic levels of our participants, such as their literacy level, and environmental factors, such as exposure to heavy metals, regarding their nutritional status, our findings showed that nutritional vulnerabilities may be present across different life stages [9,46,48]. Specifically, environmental exposures have a more pronounced effect on children’s growth, while educational background of a household exerts a more substantial influence on adults’ outcomes. This highlights the importance of integrated interventions that address environmental health risks and social determinants to foster resilience across generations.

Artisanal and small-scale mining in Mozambique is concentrated in provinces such as Manica and operates largely informally, intersecting with rural livelihoods and with limited oversight [49,50]. Unregistered mining worsens exposure risks for households dependent on local water and food systems, as was the case of our study population [50]. Our findings revealed geographical and demographic variability, with environmental risks observed among very young children. Therefore, there is a need for target environmental health interventions and research into diverse contamination pathways by soil, water, food, and air pollutants. This presents as one of the limitations of our study. The presence of mercury was demonstrated to be unexpecteda, with a negative association with stunting in children under five, contrasting with the available literature [6,9,48,51]. It is imperative that this finding be interpreted with caution, particularly given the variability of heavy metal concentrations according to demographic and dietary factors, including fish intake. It is important to acknowledge that the present study was unable to test laboratory-based heavy metal concentrations in food samples. Therefore, it is imperative that this finding should also be interpreted with caution, particularly because average heavy metal levels are below critical limits, although cumulative exposure has the potential to impair infant neurodevelopment [42,52]. In the present study, the issue of seasonal variation in heavy metals is of pertinence. Statistically, the variation is deemed to be significant, with the mean concentration falling precisely at the borderline compliance level.

Socioeconomic challenges and governance gaps related to laboratory testing and environmental and epidemiological monitoring limit detection and prevention of heavy metal exposure in mining-affected communities [9,41,50]; such was the case in the study area under consideration, which shows that an appropriate alignment of environmental management with public health priorities, such as food systems, is necessary. The complexity of the heavy metal exposure in mining settings through the food chain and air is recognized, requiring a comprehensive approach to address these matters [53]. Despite the evidence provided by the study of heavy metals in different water sources used by the population for consumption, no causal effect was determined with nutritional outcomes. This limitation can be attributed to the methodological approach and the country’s capacity to analyze different environmental matrices. Evidently, in LMICs, the monitoring of potable water and soil composition and food testing can serve as valuable instruments in the formulation and advancement of public health policies. This is especially evident in coastal and riverine regions that are dependent on fish protein, a situation that is exemplified by the area under study.

The findings of the present study demonstrated a high prevalence of stunted children, underweight adolescents, and overweight women in the Revuè sub-basin. However, no significant differences across seasons were observed. Nevertheless, it seems that participants were exposed to contaminated water, which had resulted from mining activities that discharged heavy metals into local water sources. The concentrations of arsenic and mercury in these water sources were found to exceed the international safety guidelines on a seasonal basis. This may suggest a potential risk for children’s growth and development.

## 5. Limitations

This study has limitations that should be acknowledged. Firstly, the geographical scope of the research was confined to a single basin within a single province in central Mozambique. While this approach provides valuable insights into specific local contexts, it is important to note that the findings may not be universally applicable to other regions that are characterized by distinct environmental, social, or industrial contexts. Secondly, the assessment relied exclusively on a single environmental sample (water) to estimate potential risks to public health. The absence of other environmental samples of the food chain (e.g., vegetables, fish, or cutlery), as well as biological samples (e.g., blood, urine, or hair), limits the ability to fully quantify human exposure and health outcomes, thereby constraining the interpretation of the results in terms of actual physiological impact. Thirdly, the analysis failed to consider vulnerable groups, such as miners, who are directly and occupationally exposed to mining discharges. This omission may result in an underestimation of the overall burden of exposure in populations most at risk. The aforementioned limitations underscore the necessity for subsequent research endeavors to broaden geographic coverage, incorporate biomonitoring, and encompass occupationally exposed populations. These efforts are pivotal in strengthening the evidence base for public health interventions.

## 6. Conclusions

It has been demonstrated that artisanal mining activities can contaminate water sources that are used for human consumption. This scenario poses a potential threat to public health, exerting an influence on the developmental outcomes of children and the maternal health status. Notwithstanding the limitations that have been outlined, the present study highlights the significance of environmental pollutants as a possible contributing factor to public health concerns in resource-constrained settings. The results indicate the necessity for further research to be conducted across a range of basins and provinces. In addition, the inclusion of biological sampling is recommended, as this serves as a targeted assessment of occupationally exposed populations. The enhancement of surveillance and risk assessment frameworks will be pivotal in informing policies that safeguard vulnerable communities, thereby facilitating the advancement of sustainable development goals pertaining to health, nutrition, and environmental safety in Mozambique. Consequently, it is advisable to undertake future analyses to ascertain the presence of heavy metals in agricultural and fishery products and biological samples. Consequently, there is a necessity for education and research to address this public health problem and develop coherent policies to protect vulnerable populations.

## Figures and Tables

**Table 1:** Sociodemographic characteristics of the study population (n = 1098).

Children and Adolescents (n = 587)	n (%)	Women (n = 511)	**n (%)**
**Age**		**Age**	
Children (<5 years)	345 (31.4)	Women (18–49 years)	511 (46.5)
Children (5–11 years)	99 (9.0)	**Level of Education**	
Adolescents (12–17 years)	143 (13.1)	**Women’s Ability to Read**	
**Gender**		Yes	368 (72.2)
**Female**	316 (53.8)	No	143 (27.8)
Children (<5 years)	172 (49.9)	**Husband’s Ability to Read**	
Children (5–11 years)	52 (36.1)	Yes	389 (87.2)
Adolescents (12–17 years)	92 (63.9)	No	122 (12.8)
**Male**	271 (46.2)	**Income Source**	
Children (<5 years)	173 (50.1)	Agriculture	107 (33.3)
Children (5–11 years)	47 (47.9)	Artisanal mining	37 (11.5)
Adolescents (12–17 years)	51 (52.1)	Informal/domestic	65 (20.2)
**Children’s Nutrition History**		Public/private sector	38 (11.8)
Breastfeeding period		**Family Income**	
Less than 12 months	470 (80.0)	A minimum salary *	395 (77.2)
More than 12 months	22 (3.7)	Between two minimum salaries	115 (22.5)
Food introduction		More than three minimum salaries	1 (0.2)
Before 6 months	290 (49.4)	**Income for Food**	
After 6 months	207 (35.3)	Half a minimum salary	315 (61.2)
		Less than half	165 (32.2)
		More than half	31 (6.1)
**Health Facility**		**Health Facility**	
Penhalonga	48 (8.2)	Penhalonga	58 (11.4)
Messica	164 (27.9)	Messica	133 (26.0)
Chicamba	51 (8.7)	Chicamba	105 (20.5)
Mavuzi	247 (42.1)	Mavuzi	149 (29.2)
Machipanda	77 (13.1)	Machipanda	66 (12.9)
**Season**		**Season**	
Dry	212 (36.1)	Dry	190 (37.1)
Rainy	375 (63.9)	Rainy	321 (62.7)

* Minimum salary in Mozambique varies across sectors from 99.6 to 281.3 USD; the current salary used is from April 2024, in which the public sector is 137.8 USD, the agriculture sector is 99.6 USD, artisanal fishery is 77.7 USD, and the mining sector (industrial) is 223.1 USD.

**Table 2:** Nutritional status given by indicators among children under five, disaggregated by age group, sex, season, and region (WHO Child Growth Standards).

	HA				WA				WH				MUAC				HC			
	n	<−3	<−2	Mean ± SD	n	<−3	<−2	Mean ± SD	n	<−3	<−2	Mean ± SD	n	<−3	<−2	Mean ± SD	n	<−3	<−2	Mean ± SD
**Age group**																				
12–23 mo	199	10.1	20.1	−0.7 ± 1.9	213	6.1	16.9	−0.6 ± 1.4	197	3.0	10.2	−0.4 ± 1.4	198	1.0	4.5	0.2 ± 1.3	181	7.2	17.7	−0.6 ± 1.5
24–35 mo	35	22.9	40.0	−1.1 ± 2.4	44	22.7	31.8	−1.2 ± 2.2	--	--	--	--	35	5.7	11.4	−0.4 ± 1.4	20	0	20.0	−0.5 ± 1.8
36–47 mo	25	16.0	28.0	−0.8 ± 2.7	33	15.2	21.2	−0.5 ± 2.0	--	--	--	--	12	0	16.7	−0.7 ± 1.0	9	33.3	66.7	−2.6 ± 1.6
48–60 mo	35	20.0	28.6	−1.3 ± 2.1	45	13.3	22.2	−0.7 ± 2.0	--	--	--	--	14	0	21.4	−0.7 ± 1.1	7	57.1	57.1	−1.8 ± 2.9
**Sex**																				
Male	148	14.9	28.4	−1.0 ± 2.0	169	11.2	18.9	−1.0 ± 1.7	100	5.0	12.0	1.5 ± 0.7	127	1.6	4.7	0.0 ± 1.2	106	8.5	15.1	−0.6 ± 1.7
Female	146	11.6	19.9	−0.6 ± 2.1	166	9.0	21.1	−0.4 ± 1.7	96	1.0	8.2	1.4 ± 0.9	132	1.5	9.1	−0.0 ± 1.3	111	9.9	27.0	−0.8 ± 1.7
**Season**																				
Dry	124	12.1	22.6	−0.9 ± 1.8	139	9.4	21.6	−0.8 ± 1.7	89	2.2	10.1	−0.3 ± 1.4	115	2.6	7.0	−0.1 ± 1.3	106	9.4	18.9	−0.9 ± 1.5
Rainy	170	14.1	25.3	−0.8 ± 2.2	196	10.7	18.9	−0.6 ± 1.7	108	3.7	10.2	−0.5 ± 1.5	144	0.7	6.9	0.1 ± 1.2	111	9.0	23.4	−0.6 ± 1.8

Abbreviations: SD—standard deviation. Stunting—height-for-age (HA). Underweight—weight-for-age (WA). Wasting—weight-for-height (WH). Mid-upper arm circumference (MUAC). Head circumference (HC).

**Table 3:** Nutritional status of children and adolescents, disaggregated by age group, sex, season, and region according to WHO Child Growth Standards.

			BMI-for-Age Proportion (%)				
			Severe Underweight	Underweight	Overweight	Obesity	
	Age Group	n	<−3	<−2	>+1	>+2	Mean ± SD
**Male**	6–11	21	9.5	19.8	23.8	9.5	−0.4 ± 1.7
	12–17	38	13.2	21.1	0.0	0.0	−1.0 ± 1.4
	**Subtotal**	59	11.9	20.3	8.5	3.4	−0.8 ± 1.4
**Female**	6–11	33	6.1	21.2	15.2	3.0	−0.6 ± 1.9
	12–17	72	11.1	27.8	5.6	0.0	−1.1 ± 1.5
	**Subtotal**	105	9.5	25.7	8.6	1.0	−1.0 ± 1.6
**Season**	Dry	56	3.6	21.4	16.1	0.0	−0.5 ± 1.5
	Rainy	108	13.9	25.0	4.6	2.8	−1.1 ± 1.5
	**Subtotal**	164	10.4	23.8	8.5	1.8	−0.9 ± 1.5

**Table 4:** Nutritional status of women aged 18–49 years old, disaggregated by season, region, and BMI (WHO standards).

		Proportion of Nutritional Status n (%)					
	n	Underweight (n = 22, 4.4%)		Normal Weight (n = 373, 74.3%)		Overweight and Obesity (n = 107, 21.3%)	
**Season**		Dry	Rainy	Dry	Rainy	Dry	Rainy
Total	502	2 (9.1)	20 (90.9)	144 (38.6)	229 (61.4)	42 (39.3)	65 (60.7)
**BMI**	Mean ± SD	17.0 ± 0.5	17.1 ± 1.3	22.6 ± 1.4	22.2 ± 1.7	29.8 ± 14.4	27.7 ± 3.4

**Table 5:** Multivariate regression models of nutritional status of children under five years old suffering from stunting (n = 294), with socioeconomic and environmental factors.

Predictors	Stunting n (%)	RRa (95% CI)	*p*-Value	RD (95% CI)	*p*-Value
**Gender**					
Female	85 (28.9)	1.02 (0.85–1.23)	0.72	1.01 (0.84–1.22)	0.74
Male (Ref.)	76 (25.9)	Ref.	–	Ref.	–
**Arsenic exposure**					
Risk	22 (7.5)	1.18 (0.92–1.52)	0.12	1.17 (0.91–1.50)	0.13
No risk (Ref.)	136 (46.3)	Ref.	–	Ref.	–
**Mercury exposure**					
Risk	45 (15.3)	0.76 (0.62–0.94)	**0.01**	0.77 (0.63–0.95)	**0.02**
No risk (Ref.)	113 (38.4)	Ref.	–	Ref.	–
**Food introduction**					
>6 months	84 (28.6)	0.95 (0.78–1.16)	0.64	0.96 (0.79–1.17)	0.67
<6 months (Ref.)	68 (23.1)	Ref.	–	Ref.	–

Dependent variable: stunting. Model: (intercept) gender, arsenic exposure, mercury exposure, and food introduction. Abbreviations: CI—confidence interval; Ref.—reference category; RRa—adjusted risk ratio; RD—risk difference. Bold values indicate statistical significance (*p* < 0.05). Nutritional outcomes were defined using WHO Growth Standards (2006, for children under five).

**Table 6:** Multivariate regression models of nutritional status of women with overweight (n = 107), with socioeconomic and environmental factors.

Predictors	Overweight n (%)	RRa (95% CI)	*p*-Value	RD (95% CI)	*p*-Value
**Women’s ability to read**					
No	35 (32.7)	0.98 (0.81–1.19)	0.72	0.97 (0.80–1.18)	0.75
Yes (Ref.)	72 (67.3)	Ref.	–	Ref.	–
**Husbands’ ability to read**					
No	24 (22.4)	0.74 (0.58–0.95)	**0.02**	0.75 (0.59–0.96)	**0.03**
Yes (Ref.)	69 (64.5)	Ref.	–	Ref.	–
**Family income for food**					
Half a salary	68 (63.6)	1.08 (0.89–1.31)	0.31	1.07 (0.88–1.30)	0.33
Less than half a salary	31 (28.9)	1.12 (0.91–1.38)	0.17	1.11 (0.90–1.37)	0.16
More than half a salary (Ref.)	8 (7.5)	Ref.	–	Ref.	–
**Arsenic exposure**					
No risk	107 (100)	–	–	–	–
Risk (Ref.)	0 (0.0)	Not estimable	–	Not estimable	–
**Mercury exposure**					
Risk	19 (17.8)	0.95 (0.78–1.16)	0.75	0.96 (0.79–1.17)	0.74
No risk (Ref.)	88 (82.2)	Ref.	–	Ref.	–

Dependent variable: overweight. Model: (intercept) women’s ability to read, husbands’ ability to read, family income for food, arsenic exposure, and mercury exposure. Abbreviations: CI—confidence interval; Ref.—reference category; RRa—adjusted risk ratio; RD—risk difference. Bold values indicate statistical significance (*p* < 0.05).

**Table 7:** Chemical analysis findings of Revuè River sampling points (Penhalonga, Messica, Chicamba, and Mavuzi) and standards values during the dry and rainy seasons in 2025.

Parameters		Dry Season				Rainy Season					
	Sampling Points	Penhalonga	Messica	Chicamba	Mavuzi	Penhalonga	Messica	Chicamba	Mavuzi	WHO Std. [37]	National Std. [32]
Temperature (°C)		28.3	24.0	24.0	25.2	25.7	26.0	27.7	28.0	-	-
pH		7.3	6.7	7.0	7.1	7.3	7.0	7.7	7.7	6.5–8.5	6.5–8.5
Turbidity (NTU)		3.5 2.6	**7.2** 0.2	**160.0** **5.3**	**97.9** 0.6	**864.0** 1.0	**807.0** 0.0	**51.5** **7.0**	**209.5** 2.0	5	5
		*Mean ± SD*	*Mean ± SD*	*Mean ± SD*	*Mean ± SD*	*Mean ± SD*	*Mean ± SD*	*Mean ± SD*	*Mean ± SD*	*Mean*	*Mean*
As (mg/L)	Surface river water	**0.073 ± 0.004**	**0.034 ± 0.008**	0.005 ± 0.000	0.005 ± 0.000	0.005 ± 0.000	0.004 ± 0.000	0.005 ± 0.000	0.004 ± 0.000	0.01	0.01
	Deep river water	**0.638 ± 0.695**	**0.037 ± 0.008**	0.005 ± 0.000	**0.022 ± 0.024**	0.003 ± 0000	0.004 ± 0.000	0.005 ± 0.000	0.004 ± 0.000		
	Groundwater	0.005 ± 0.000	0.005 ± 0.000	0.005 ± 0.000	0.005 ± 0.000	0.005 ± 0.000	0.005 ± 0.000	0.004 ± 0.000	0.005 ± 0.000		
Cd (mg/L)	Surface river water	0.003 ± 0.000	0.003 ± 0.000	0.003 ± 0.000	0.003 ± 0.000	0.003 ± 0.000	0.003 ± 0.000	0.003 ± 0.000	0.003 ± 0.000	0.003	0.003
	Deep river water	0.003 ± 0.000	0.003 ± 0.000	0.003 ± 0.000	0.003 ± 0.000	0.003 ± 0.000	0.003 ± 0.000	0.003 ± 0.000	0.003 ± 0.000		
	Groundwater	0.003 ± 0.000	0.003 ± 0.000	0.003 ± 0.000	0.003 ± 0.000	0.003 ± 0.000	0.003 ± 0.000	0.003 ± 0.000	0.003 ± 0.000		
Hg (mg/L)	Surface river water	**>0.5**	**>0.5**	**0.005 ± 0.000**	**0.005 ± 0.001**	**0.005 ± 0.001**	**0.005 ± 0.000**	**0.005 ± 0.001**	**0.005 ± 0.001**	0.006	0.001
	Deep river water	**>0.5**	**>0.5**	**0.004 ± 0.001**	**0.004 ± 0.000**	**0.004 ± 0.000**	**0.004 ± 0.000**	**0.003 ± 0.000**	**0.004 ± 0.000**		
	Groundwater	**0.005 ± 0.000**	**0.005 ± 0.000**	**0.005 ± 0.001**	**0.003 ± 0.000**	**0.004 ± 0.001**	**0.005 ± 0.000**	**0.003 ± 0.000**	**0.004 ± 0.001**		
Pb (mg/L)	Surface river water	0.005 ± 0.000	0.005 ± 0.000	0.005 ± 0.000	0.005 ± 0.000	0.005 ± 0.000	0.005 ± 0.000	0.005 ± 0.000	0.005 ± 0.000	0.01	0.01
	Deep river water	0.005 ± 0.000	0.005 ± 0.000	0.005 ± 0.000	0.005 ± 0.000	0.005 ± 0.000	0.005 ± 0.000	0.005 ± 0.000	0.005 ± 0.000		
	Groundwater	0.005 ± 0.000	0.005 ± 0.000	0.005 ± 0.000	0.005 ± 0.000	0.005 ± 0.000	0.005 ± 0.000	0.005 ± 0.000	0.005 ± 0.000		

Abbreviations: NTU—nephelometric turbidity unit; SD—standard deviation; std.—standard values. Bold values are above these standards. Heavy metals analyzed: As—arsenic, Cd—cadmium, Hg—mercury, and Pb—lead.

**Table 8:** Mean heavy metal concentrations compared to national and international standards and seasonal variation.

Parameter (mg/L)	Water Sampling Points	Combined Mean Concentration	National Standards * [33]	WHO Standards ** [38]	Seasonal Variation (*p*-Value)
**Arsenic (As)**	River (surface and deep)	0.01	0.01	0.01	**<0.001**
	Groundwater	<limit of detection	0.01	0.01	--
**Cadmium (Cd)**	River and groundwater	<0.003	0.003	0.003	**<0.001**
**Mercury (Hg)**	River (surface/deep)	0.001	0.001	0.006	**<0.001**
**Lead (Pb)**	River and groundwater	0.01	0.01	0.01	**<0.001**

Abbreviations: * standard value in the country; ** World Health Organization standards. Bold values are statistically significant (*p* < 0.05).

## Data Availability

Data generated during the study, as well as the datasets, will be available from the Fernando Pessoa University and the National Institute of Health of Mozambique servers after the conclusion of the project, as well as for further analysis upon request to the Principal Investigator.

## Abbreviations

The following abbreviations are used in this manuscript:

As	Arsenic
Cd	Cadmium
BA	BMI-for -age
BMI	Body mass index
PAF	Population attributable fraction
HA	Height-for- age
HC	Head circumference-for-the-age
Hg	Mercury
HQ	Hazard quotient
HW	Height-for- weight
MUAC	Middle-upper arm circumference
mo	Months
NTU	Nephelometric turbidity unit
Pb	Lead
RD	Risk difference
Ref.	Reference category
RfD	Reference dose
RR	Relative risk
SD	Standard deviation
SPSS	Statistical Package for Social Science
WA	Weight-for- age
WHO	World Health Organization
WH	Weight-for- height
